# A Comprehensive Computational NMR Analysis of Organic Polyarsenicals including the Marine Sponge-Derived Arsenicins A–D and Their Synthetic Analogs

**DOI:** 10.3390/md21100511

**Published:** 2023-09-27

**Authors:** Andrea Defant, Ines Mancini

**Affiliations:** Laboratory of Bioorganic Chemistry, Department of Physics, University of Trento, Via Sommarive 14, I-38123 Trento, Italy

**Keywords:** marine metabolite, polyarsenical, structural characterization, calculated NMR spectrum, chemical shift, density functional theory, GIAO, CSGT

## Abstract

The adamantane structure of arsenicin A and nor-adamantane structures of arsenicins B–D have gained attention as unique natural polyarsenicals, as well as hits showing promising anticancer activity. The purpose of this study is to apply the predictive power of NMR DFT calculations in order to identify a valid tool to be used in the structural elucidation of similar molecules. ^1^H- and ^13^C-NMR chemical shifts of twelve natural and synthetic polyarsenical analogs were calculated and validated by comparison with experimental data acquired in CDCl_3_ solutions, in regard to mean absolute error (MAE) values under various combinations of two methods (GIAO and CSGT), four functionals and five basis sets, also considering relativistic effects. The best computational approaches are highlighted for predicting the chemical shifts of ^1^H and ^13^C nuclei and *J*(^1^H,^1^H) coupling constants in the series of O- and S-polyarsenicals. This comprehensive analysis contributes to making NMR spectroscopy appealing for the structural elucidation of such molecules, contrary to the first structural elucidation of natural arsenicin A, in which the experimental NMR analysis was limited by the poor presence of proton and carbon atoms in its structure and by the shortage of reference data.

## 1. Introduction

Liquid solution nuclear magnetic resonance (NMR) is a powerful and widely used experimental technique in the structural study of organic molecules. Moreover, it is a pivotal spectroscopic method used in conventional protocols to characterize even very complex structures of natural products, mainly represented by secondary metabolites whose study is largely still interesting for the discovery of novel biologically active molecules [1]. NMR calculations could provide a useful tool to distinguish between closely related structures, and to confirm or revise the original structures assigned using extensive experimental NMR spectroscopy [2].

Previously, NMR calculations were mostly carried out using density-functional theory (DFT), which is a promising alternative to conventional ab initio methods in quantum chemistry. DFT methods were successful in predicting various molecular properties, including magnetic response properties and shielding tensors. Moreover, their considerable accuracy usually provides significant results at a relatively low computational cost. Since the first studies in the 1990s, a number of successful applications were reported on a wide variety of organic, organometallic and inorganic species, ranging from natural products to metal complexes [3].

Besides ^1^H- and ^13^C-NMR chemical shifts, another piece of diagnostic information to establish the structural connectivity in organic molecules is given by the coupling constants *J*(^1^H,^1^H) and *J*(^1^H,^13^C). However, experimental *J*(^1^H,^13^C) are seldom known because the spectra are mostly acquired under proton-decoupled conditions. Therefore, focusing on ^1^H and ^13^C as NMR-active nuclei, NMR calculation can predict the corresponding chemical shifts and their coupling constants, to be compared with experimental data.

**Figure 1:**
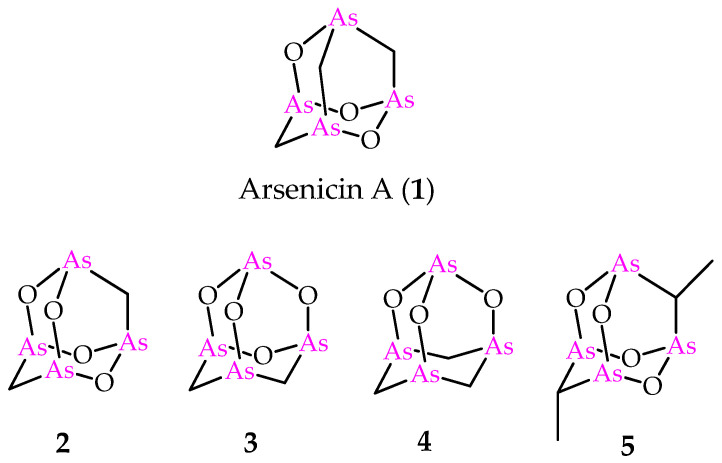
Molecular structures of natural arsenicin A (**1**) and its synthetic adamantane cage analogs **2**–**5**.

In 2006, one of us and coauthors reported arsenicin A (Figure 1), isolated from the poecilosclerid sponge *Echinochalina bargibanti* collected along the north-eastern coast of New Caledonia, as a peculiar case in the natural products scenario. In fact, if organic monoarsenicals were known as metabolites specifically of marine origin, arsenicin A was the first polyarsenic organic compound from Nature [4]. Deceptively simple NMR spectra (two and three signals in ^13^C- and ^1^H-NMR spectra, respectively) did not at all correspond to the ease of identifying its adamantane-type structure and molecular formula C_3_H_6_As_4_O_3_. The failure to obtain suitable crystals from the minimum amount of the isolated compound was combined with the scarcity of known NMR data currently available for organoarsenicals. Furthermore, the limited amount available for arsenicin A, as in general from the isolation of almost all secondary metabolites of marine origin, prevented the ^75^As-NMR analysis based on a moderately sensitive quadrupolar spin 3/2 nucleus that usually produces broad lines over a wide chemical shift range. These obstacles were successfully overcome by the application of IR spectroscopy. In detail, the comparison of DFT-calculated IR spectra for a series of putative structures with the experimental IR spectrum of the natural product provided a very good agreement with only one of them [4]. Moreover, experimental and theoretical vibrational analysis including IR and Raman spectroscopies emerged as a valid tool in the structural study of this class of molecules and reinforced the role of these techniques when NMR can be scarcely reliable [5]. Later, the correct assignment based on this method was further supported by the crystal structure of synthetic arsenicin A [6].

Increased interest was focused on arsenicin A due to its potent antibacterial activity [4] and the strong in vitro activity against acute promyelocytic leukemia (PML) cell line; the latter provided better results than arsenic (III) oxide, and was approved in 2000 as a therapeutic agent by the Food and Drug Administration (FDA) [7]. Furthermore, a fast synthetic procedure provided an efficient access to a series of arsenicin A analogs (Figure 1) [8]. Additionally, in this case, the molecular structures of compounds **2**–**4** were endorsed by the comparison of experimental and calculated IR spectra. These products were used for in vitro screening on the National Cancer Institute (NCI)-USA full cancer line panel, and each tested compound was more active than arsenic (III) oxide [8]. The biological evaluation included also the dimethyl compound **5** (Figure 1), first reported by Keppler and coworkers, with its structure validated by x-ray data [9]. Very recently, the polyarsenical **5** and its diethyl and dipropyl analogs, made available via a selective synthesis starting from arsenic trioxide, the suitable carboxylic acid and the corresponding anhydride, have been evaluated as inhibitors of glioblastoma stem cells, and are recognized as a promising therapeutic target in glioblastoma treatment. By showing submicromolar GI_50_ values and high selectivity toward non-tumor cell lines, these alkyl polyarsenicals are very promising for further biological evaluation [10].

In 2008, one of us and coauthors reported a computational NMR analysis of a series of arsenicals, ranging from simple models to the target arsenicin A. The structure of this metabolite is indeed intriguing and interesting from a computational point of view, since it is small, non-polar and conformationally rigid. The DFT calculations for twelve potential isomeric structures actually provided only the reported structure of arsenicin A as fully consistent with experimental NMR spectra [11].

**Figure 2:**
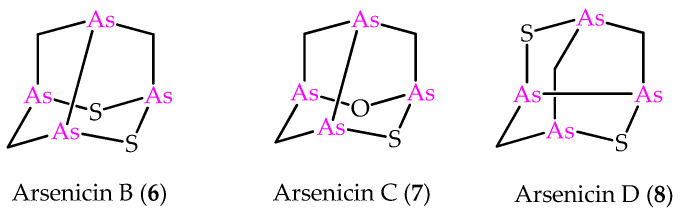
Molecular structures of minor metabolites isolated from the same New Caledonian sponge *Echinochalina bargibanti*.

In the light of the predictive power of NMR DFT calculations applied to the arsenicin A structure, the method was later applied to the structural elucidation of the minor metabolites arsenicin B and arsenicin C (**6** and **7** in Figure 2), isolated from the same sponge extract containing arsenicin A [12]. In detail, combining high resolution mass spectrometric data and extensive NMR analysis enabled the assignment of arsenicin B’s structure, whereas the low quantity of the isolated arsenicin C prevented the full structural definition, which was accessible via NMR calculations on a series of putative structures. The molecules **6** and **7** are peculiar in showing a sulfur-containing nor-adamantane cage, characterized by an unusual As–As bonding. Additionally, the very scarce metabolite named arsenicin D (**8**, Figure 2) has recently been identified as a diasteroisomer of arsenicin B by comparison with the reported synthetic polyarsenical whose crystal structures was determined by X-ray diffraction [13].

**Figure 3:**
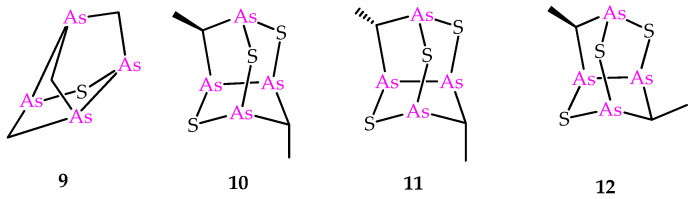
Molecular structures of known synthetic sulfur-containing polyarsenicals **9–12**.

Besides the compounds **6** and **8**, the sulfur-containing polyarsenical **9** (Figure 3) was obtained by reacting arsenicin A with aqueous sodium sulfide, its structure confirmed by single-crystal X-ray diffraction data and evaluated as an acute PML inhibitor, proving to be more active than arsenicin A [13]. The products **10**–**12** (Figure 3) have recently been obtained from the dimethyl analog **5** via a similar procedure, and characterized using NMR DFT calculations. However, their very poor water solubility, observed to be affected by replacing oxygen with sulfur atoms in the structural cages, prevented the biological evaluation of these polyarsenicals through in vitro screening [10].

Fifteen years after the first NMR calculation was applied to arsenicin A’s structure [11], we now report a comprehensive study on DFT-calculated NMR analysis of the whole series of arsenicin-like polyarsenicals, which in the meantime is enriched by natural and synthetic analogs of promising interest for their antitumor activities [14]. Aiming at filling the gap in the NMR report for polyarsenicals, the present work includes the calculations of ^1^H- and ^13^C chemical shifts, and ^1^H,^1^H coupling constants in terms of both functionals and basis sets, validated by comparison with experimental data. The purpose is to establish a DFT protocol for the NMR analysis of these adamantane and sulfur-containing nor-adamantane cage molecules, identifying the most suitable types of functionals and basis sets, so as to offer a valid approach in the structural elucidation of further structures belonging to the chemical space of the organic polyarsenic cage. This approach falls within a more general topic, recently pointed out by Bally and Rablen [15]. These authors state that, for a given set of molecules, there are few comparisons on the performance of the various methods available so far to establish which procedures provide the best agreement with experiment at the lowest computational cost.

## 2. Results and Discussion

Our interest has been focused on the computational analysis of NMR data of polyarsenicals, peculiar for the intriguing rigid cage of the structures of arsenicins A–D (Figure 1 and Figure 2). In our study we have included also the dimethyl compound **5** as a representative example of alkyl analogs, but not the known diethyl and dipropyl arsenicals [10] which would have involved the evaluation of the possible conformations on the alkyl chain.

Each structure’s geometry was minimized by using B1B95/6-311+G(3df,2pd) as a combination of electronic correlation functional and basis set, in chloroform, via a Conductor-like Polarized Continuum Model (C-PCM), obtaining no vibrational imaginary wave number modes as indication of a reached minimum in the potential energy surface.

The NMR methods applied in our study were the gauge-including atomic orbital (GIAO) [3] and the continuous set of gauge transformation (CSGT) [16], which are two of the most common approaches for calculating nuclear magnetic shielding tensors. GIAO is the most reported in the literature, and the results obtained by its application are often more accurate than those calculated with other approaches, at the same basis set size [17]. The CSGT method is more accurate in some cases, but requires large basis sets [18].

The effect of considering solvent can affect computed molecular geometries and the calculation of shielding constants. However, the commonly used NMR solvent CDCl_3_ has a negligible role [2].

Different combinations of functionals and basis sets were considered. Regarding the choice of functional, we followed Perdew’s “Jacob’s ladder”, which reports in a hierarchical classification the density approximations for the exchange-correlation energy. Based on an increasing accuracy, we used OLYP, which is a type of generalized gradient approximation (GGA); M06-L, which is a meta-generalized gradient approximation (M-GGA), PBE1PBE, a hybrid generalized gradient approximation (H-GGA); and TPSSh, which is a hybrid meta-generalized gradient approximation (HM-GGA) [19]. It is of note that we could not employ double hybrid functionals by using Gaussian software, because it is not able to perform NMR calculations with this type of functionals.

The choice of the basis set is also able to affect the results’ accuracy. The data reported herein derive from the employment of five basis sets: aug-cc-pVDZ, aug-cc-pVTZ, def2-TZVPP, pc-Sseg-2, x2c-TZVPPAll-s, with the last one considering the relativistic effects.

The relativistic effects are provided by the presence of one or more heavy atoms on the NMR shifts of its neighboring atoms. In the previous study, a relativistic approach was applied using ZORA (zeroth-order regular approximation) formalism to discriminate the effective structure of arsenicin A among a series of eligible isomers [11]. However, arsenic (Z = 33) is borderline in adopting both types of basis set, as suggested by the definition of heavy atoms when Z is higher than 36 [20].

In the selection of functionals/basis sets, a wider approach was adopted, in which calculations with different functionals and basis sets, even higher, were considered. In detail, the basis sets tested on arsenicin A as a model for oxygen-containing adamantane cage structures and arsenicin B as model for the sulfur-containing series included B3LYP, CAM-B3LYP, B3PW91, BHandHLYP, HSEh1PBE, mPW1PW91, ωB97xD, B97-2, B1B95, M05, M06, M06-HF, M06-2X, LSDA and AFPD, each of them combined to one or more different basis sets, without relativistic effects (pc-2, pcseg2, pcSseg-0, pcSseg-1, pcSseg-3, aug-pcSseg-2, def2-SVPP, def2-QVPP, Sapporo-DZP-2012-diffuse, Jorge-TZP, Jorge-TZP-DKH, Sadlej-pVTZ) and taking into account relativistic effects (ANO-DK3, NMR-DKH(TZ2P), x2c-SVPall-s and x2c-QVPPall-s). The latter ones performed similarly or often worse than those selected in our study, even taking longer calculation times (unreported data). This behavior is in line with the reported evidence that an increasing basis set requiring more computational time would not necessarily provide more accurate chemical shifts [2].

### 2.1. DFT-Calculated vs. Experimental ^1^H-NMR Chemical Shifts of Polyarsenicals ***1***–***12***: Evaluation of Functionals and Basis Sets

**Figure 4:**
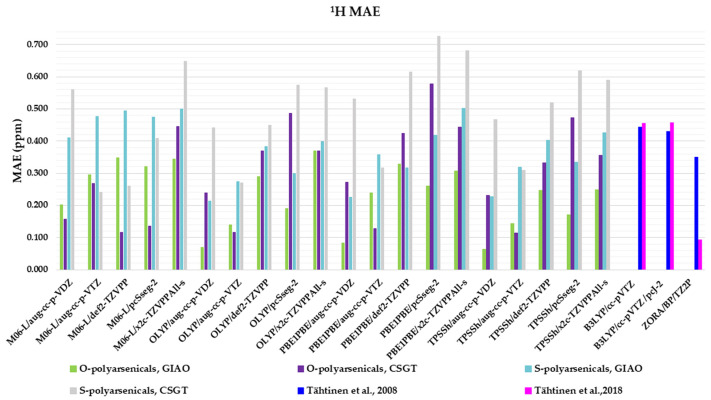
Mean absolute errors (MAEs) for ^1^H nuclei obtained using the indicated functional/basis set combinations for O-polyarsenicals **1**–**5** and S-polyarsenicals **6**–**12**, in comparison with reported data for arsenicin A (**1**) and **5** (blue bar) [11], and arsenicin B (**6**) and arsenicin C (**7**) (magenta bar) [12].

Four functionals belonging to different categories, five basis sets and both GIAO and CSGT methods were selected to be used in the calculations of the chemical shifts in chloroform. The full data obtained are reported in Table S1, in comparison with the experimental values of polyarsenicals in CDCl_3_. The corresponding mean absolute errors (MAEs) are presented in Figure 4, taking as references the known computational data for arsenicin A (**1**) and dimethyl analog **5** [11], and sulfur-containing arsenicin B (**6**) and arsenicin C (**7**) [12].

Regarding the O-polyarsenicals **1**–**5**, the most used GIAO method works better than CSGT when a small basis set (aug-cc-p-VDZ) is combined also to different functionals. In detail, the MAE values were 0.069 with OLYP, 0.084 with PBE1PBE and 0.063 ppm with TPSSh were obtained. It is of note that each result obtained with the GIAO method and almost all of those obtained with the CSGT method are better than the values reported for molecules **1** and **5** [11]. Additionally, when a wider basis set is applied (i.e., aug-cc-p-VTZ vs. aug-cc-p-VDZ), CSGT performed similarly to the GIAO method. However, it must be taken into account that using a wider basis set is more time-consuming. The relativistic basis set x2c-TZVPAll-s does not improve the chemical shift values, even combined to different functionals (M06-L, OLYP, PBE1PBE and TPSSh). This can be deduced by MAEs in the range 0.25–0.37 ppm.

Additionally, for the S-polyarsenicals **6**–**12**, a wide spectrum of results was observed. In detail, GIAO is generally better than the CSGT method. The most favorable MAEs are obtained by using the same small basis sets and the same functionals applied for O-polyarsenicals, whereas the functional M06-L gives the worst data, in the range 0.4–0.5 ppm. The best result corresponds to a 0.21 ppm from the combination OLYP/aug-cc-p-VDZ. By considering the relativistic basis sets, MAE values in the range 0.40–0.50 ppm are achieved. It is noteworthy that the best value (0.12 ppm) reported for natural sulfur-containing arsenicals is provided by the relativistic zeroth-order regular approximation (ZORA) [12].

The high-quality of these data is evident if we refer to the average errors of up to 0.4 ppm or more for ^1^H shifts by NMR chemical shift calculations, even when performed using some of the best computational methods [2].

**Figure 5:**
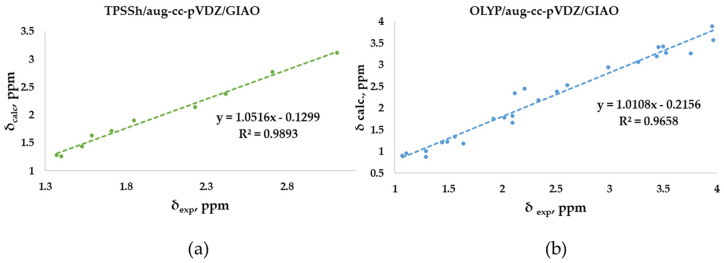
Best correlation plots of calculated (by the best method as indicated) vs. experimental ^1^H- NMR chemical shifts for (**a**) O-polyarsenicals **1–5** and (**b**) S-polyarsenicals **6–12**.

The linear regression procedure is a criterion for verifying whether experimental chemical shifts have been reproduced without random errors in the computational method. Meaningful indications are deduced based on how far the slope of the correlation line is from unity and the intercept from zero [2]. In our study, the experimental chemical shifts measured in CDCl_3_ for the O-adamantane cage structures **1**–**5** (Table S1) were correlated to the corresponding calculated values, showing the linear correlation R^2^ coefficients (Figure 5a). Our results show an R^2^ coefficient of 0.9893, indicating a good linear correlation. Similarly, the correlation between measured and calculated chemical shifts has been considered for the sulfur-containing structures **6**–**12** (Figure 5b). In this case, a lower R^2^ coefficient of 0.9658 was obtained. This behavior could be attributed to the fact that the methyl protons are not considered magnetically equivalent in the calculation, as in the experimental measurement. In fact, as highlighted in Figure 5b, the values that deviate the most from the linear fit are those associated with these methyl protons, of which an arithmetic average was considered. In support of this hypothesis, a higher R^2^ (0.9813) was obtained by eliminating these values from the correlation plot. Moreover, both correlations were derived by using the same relatively small basis set (double-zeta), a further example of how an increasing basis sets does not necessarily provide more accurate results [2].

### 2.2. Calculation of ^1^H,^1^H Coupling Constants

Coupling constants for structures **1**–**12** were calculated and the results are reported in Table 1. We used the functional/basis set combinations giving the best data in terms of MAEs. In detail, for O-polyarsenicals **1**–**5,** the same TPSSh functional was coupled with basis sets selected in order to increase the performance (from aug-cc-pVDZ to aug-cc-pVTZ) and to take into account the relativistic effects (x2c-TZVPPAll-s). Regarding the S-polyarsenicals **6**–**12**, the OLYP functional was associated with the same basis sets. A very good agreement was observed for experimental and calculated geminal *J* values of molecules **1**, **3** and **4**, and of sulfur-containing polyarsenicals **6**–**12** when the triple-zeta basis set was used; moreover, this was also true when the relativistic x2c-TZVPPAll-s basis set was applied.

A peculiar result was observed for molecule **2**, for which the calculated geminal coupling constant (Table 1) disagreed with the experimental observation of a lone singlet at 1.85 ppm in the ^1^H-NMR spectrum, correlated to 30.60 ppm in the heteronuclear single quantum coherence (HSQC) experiment. The structural assignment was supported by high resolution mass spectrometry and extensive vibrational analysis, including the comparison of FT-IR spectrum with DFT-calculated IR frequencies [8], and Raman spectroscopy [21]. The structure belongs to the D_2d_ group point and this high symmetry gives a ^1^H singlet, as reported for other rigid molecules, showing the same symmetry [22]. Of note, a result reproducing the experimental evidence has never been obtained by us, also applying symmetry constrain and taking into account the paramagnetic and diamagnetic contribution 

### 2.3. DFT-Calculated vs. Experimental ^13^C-NMR Chemical Shifts Polyarsenicals ***1***–***12***: Evaluation of Functionals and Basis Sets

**Figure 6:**
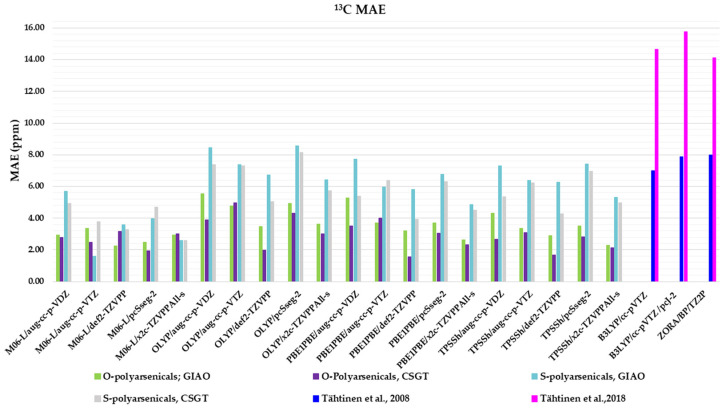
Mean absolute errors (MAEs) for ^13^C nuclei obtained using the indicated functional/basis set combinations for O-polyarsenicals **1**–**5** and S-polyarsenicals **6**–**12**, in comparison with reported data for arsenicin A (**1**) and **5** (blue bar) [11], and arsenicin B (**6**) and arsenicin C (**7**) (magenta bar) [12].

The same combinations of functional/basis set/method adopted for ^1^H-NMR evaluation are here applied to compare ^13^C-NMR-calculated chemical shifts with the experimental data (Table S2). The corresponding MAEs are reported in Figure 6.

Regarding the O-polyarsenicals **1**–**5**, the CSGT method was better than GIAO. The minor MAE values were derived from PBE1PBE/def2-TZVPP, TPSSh/def2-TZVPP, OLYP/def2-TZVPP and M06-L/pcSseg-2 (1.55, 1.66, 1.97 and 1.95 ppm, respectively). The GIAO method provides MAEs within the range 2.26–5.55 ppm. It is evident that all these data are better than those calculated for natural arsenicin A and the dimethyl analog **5**, which were previously reported with values in the range 14.1–15.8 ppm [11].

The performance of the CSGT and GIAO methods was comparable when applied to the S-polyarsenicals **6**–**12**. In detail, the most favorable MAE was obtained through M06-L/aug-cc-p-VTZ using the GIAO method, whereas values ranging from 2.59 and 8.43 ppm are provided by all other computational conditions. These data are much better than the ones showing a variability in the range 14.6 and 16.2 ppm, reported in a previous study [12].

Additionally, they can be evaluated in comparison with average errors of up to 10 ppm or more obtained for ^13^C shifts calculated even using some of the best computational methods [2].

**Figure 7:**
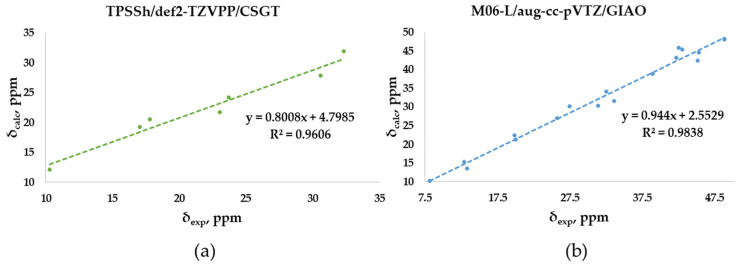
Best correlation plots of calculated (by the indicated method) vs. experimental ^13^C-NMR chemical shifts for (**a**) O-polyarsenicals **1–5** and (**b**) S-polyarsenicals **6–12**.

The experimental chemical shifts measured in CDCl_3_ for the O-adamantane cage structures **1**–**5** (Table S2) are related to the corresponding calculated values. The correlation shows a linear fit associated with the best, although low enough, R^2^ value (0.9606) obtained through TPSSh/def2-TZVPP/CSGT (Figure 7a), although PBE1PBE/def2-TZVPP/CSGT gives a minor MAE value (1.66 vs. 1.55 ppm). The correlation between measured and calculated values for the S-polyarsenicals **6**–**12** provides instead a good R^2^ coefficient (0.9838), as reported in Figure 7b. The smaller number of points (7) could have a decisive role in lowering the R^2^ value obtained for O-polyarsenicals, as supported by the comparison with the case of S-polyarsenicals, where the linear plot takes into account 17 different ^13^C-NMR signals.

### 2.4. Comprehensive Evaluation of ^1^H- and ^13^C- Chemical Shifts

**Figure 8:**
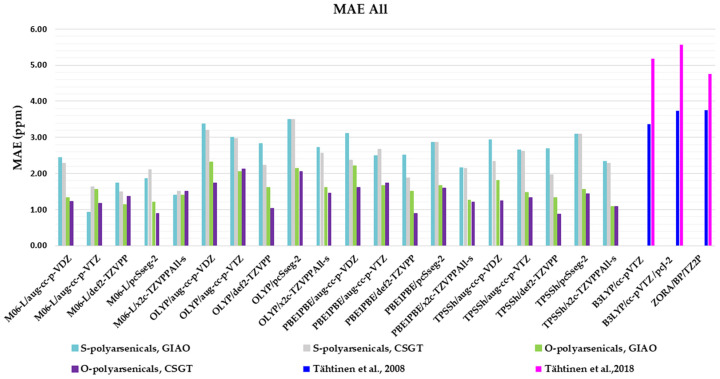
Mean absolute errors (MAEs) for both ^1^H and ^13^C nuclei obtained using the indicated functional/basis set combinations for O-polyarsenicals **1**–**5** and S-polyarsenicals **6**–**12**, in comparison with reported data for arsenicin A (**1**) and **5** (blu bar) [11], and arsenicin B (**6**) and arsenicin C (**7**) (magenta bar) [12].

The evaluation described so far has specifically considered ^1^H- and ^13^C-NMR chemical shifts of the O- and S-polyarsenical classes. Figure 8 summarizes the whole MAE values and it can be useful to select the best compromise method for evaluating both NMR-active nuclei. In detail, the data for the O-series including the compounds **1**–**5** are in the region 0.88–2.33, with the lowest values (0.88 ppm) for M06L/pcSseg-2/CSGT, as well as for TPSSh/def-2TZVPP/CSGT. The known MAEs for **1** and **5** ranged from 3.36 to 3.75 ppm [11]. The data for the S-series (**6**–**12**) are between 0.94 and 3.51 ppm, with the best value of 0.94 ppm for M06-L/aug-cc-p-VTZ/GIAO. They must be compared with the values in the range 4.77–5.56 reported in the case of S-polyarsenicals **6**–**8** [12] and **10**–**12** [10].

**Figure 9:**
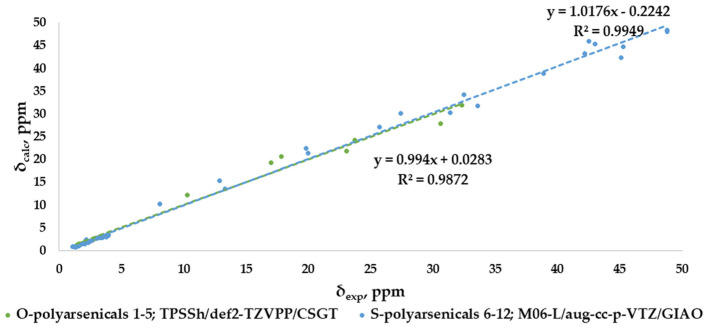
Best correlation plots of combined ^1^H-NMR and ^13^C-NMR chemical shifts calculated (by the indicated method) vs. experimental for O-polyarsenicals **1**–**5** and S-polyarsenicals **6**–**12**.

The correlation plots for combined ^1^H- and ^13^C-NMR chemical shifts are shown in Figure 9 for each selected result according to the best MAE value (0.88 and 0.94 ppm, respectively), with good and very good R^2^ values, specifically 0.9872 for O-polyarsenicals and 0.9949 for S-polyarsenicals.

## 3. Materials and Methods

### Computational Methods

DFT calculation was preoptimized in gas phase and later performed in chloroform by using a Conductor-like Polarized Continuum Model (C-PCM) [23]. Calculations were carried out on a PC running at 3.4 GHz on an AMD Ryzen 9 5950X 16-core (32 threads) processor with 32 GB RAM and 1 TB hard disk with Windows 10 Home 64-bit as an operating system. The structures of compounds were built using GaussView 6.0, and the Gaussian program, 16w version 1.1 [24] was used in the geometry optimization at a density functional theory (DFT) level of theory. The optimized geometry was obtained by using RFO step, integral precision = superfine grid and type convergence criteria, and invoking gradient employing 6-311+G(3df,2pd) basis set for all atoms. The electronic correlation functional B1B95, where the gradient-corrected DFT with Becke hybrid functional B1 [25] for the exchange part and the B95 for correlation function [26] was utilized. The optimized structural parameters were taken in the vibrational energy calculations at the DFT levels to characterize all stationary points as minima. No imaginary wave number modes were obtained for the optimized structure (Table S3), proving that a local minimum on the potential energy surface was actually found.

NMR simulation was carried out in chloroform, employing all the combinations of five different basis sets: aug-cc-pVDZ, aug-cc-pVTZ, def2-TZVPP, pcSseg-2 and x2c-TZVPPAll-s basis set [27] and four different functionals: the generalized gradient approximation (GGA) functional OLYP [28,29,30,31], the hybrid generalized gradient approximation (H-GGA) functional PBE1PBE also known as PBE0 [32], the meta-generalized gradient approximation (M-GGA) M06L functional [33,34] and the hybrid meta-generalized gradient approximation (HM-GGA) TPSSh functional [35]. Magnetic properties were calculated with GIAO [3,16,36,37,38,39] and CSGT [3,16] schemes. For *J*(^1^H,^1^H) calculations, the indirect nuclear spin−spin coupling constant (SSCC) was calculated using three different basis sets (aug-cc-pVDZ, aug-cc-pVTZ and x2c-TZVPPAll-s) in combination with exchange and electronic correlation functional TPSSh for O-polyarsenicals or OLYP for sulfur-containing polyarsenical molecules. Only the Fermi contact (FC) contribution using Gaussian (keyword onlyFC) was considered.

The isotropic shift constants (σ) were obtained for each nucleus and these were converted to a chemical shift (δ) value according to equation: δ_i_ = σ_TMS_ − σ_i_. The reference substance was tetramethylsilane (TMS), calculated at the same level of theory.

## 4. Conclusions

An in-depth NMR computational analysis was applied to the whole series of polyarsenicals showing the cage structures of natural arsenicins A–D reported so far, in order to suggest a convenient and consistent protocol. The performance of various combinations of methods (GIAO and CSGT), functionals (four) and basis sets (five) in computing ^1^H and ^13^C chemical shifts, and *J*(^1^H,^1^H) in chloroform has been evaluated. It was then validated by comparison with experimental data of twelve natural and synthetic molecules and DFT-calculated spectra that had been previously described for a reduced number of them. The most suitable conditions were identified as (i) GIAO/TPSSh/aug-cc-pVDZ for ^1^H shifts in O-polyarsenicals; (ii) GIAO/OLYP/aug-cc-pVDZ for ^1^H shifts in S-polyarsenicals; (iii) the triple-zeta basis sets, including also the relativistic x2c-TZVPPAll-s, for obtaining a very good agreement in most compounds between experimental and calculated *J* values; (iv) CSGT/TPSSh/def2-TZVPP for ^13^C shifts in O-polyarsenicals and (v) GIAO/M06L/aug-cc-pVTZ for ^13^C shifts in S-polyarsenicals. Based on this comprehensive study on the best prediction method, NMR analysis can now be applied with good reliability to offer a valid approach in the structural elucidation of further similar polyarsenicals, motivated by the proven interest in this class of compounds due to their promising biological activities.

## Figures and Tables

**Table 1:** Experimental and calculated *J*(^1^H,^1^H) values [Hz] for polyarsenicals **1–12**. ^1^ Arbitrary numbering of atoms as reported on the structures in Figure S1.

		O-Polyarsenicals			
			TPSSh		
Comp.	Expt ^2^		aug-cc-pVDZ	aug-cc-pVTZ	x2c-TZVPPAll-s
**1**	^2^*J* = 13.8	^2^ *J*	−15.3	−13.2	−13.9
	^4^*J* = 0.9	^4^ *J*	1.1	1.6	1.6
**2**	n.d.	^2^ *J*	−15.0	−13.0	−13.5
**3**	^2^*J* = 13.8	^2^ *J*	−15.2	−13.1	−13.7
**4**	^2^*J* = 13.8	^2^ *J*	−15.2	−13.1	−13.7
**5**	*J*_CH,Me_ = 7.9	*J*_CH,Me_	7.1	8.1	8.3
		**S-polyarsenicals**			
			**OLYP**		
			**aug-cc-pVDZ**	**aug-cc-pVTZ**	**x2c-TZVPPAll-s**
**6**	^2^*J* = 12.4	*J*_14,15_	−13.6	−12.0	−12.6
	^2^*J* = 13.5	*J*_12,13_	−14.2	−12.9	−13.5
	^2^*J* = 13.8	*J*_10,11_	−14.4	−13.1	−13.7
	^4^*J* = 1.7	*J*_10,12_	0.76	1.1	1.1
**7**	^2^*J* = 12.8	*J*_14,15_	−13.8	−12.3	−12.9
	^2^*J* = 13.7	*J*_12,13_	−14.3	−13.0	−13.6
	^2^*J* = 13.8	*J*_10,11_	−14.4	−13.0	−13.6
	^4^*J* = 1.7	*J*_13,15_	0.70	1.1	1.1
	^4^*J* = 1.9	*J*_10,12_	0.92	1.4	1.3
**8**	^2^*J* = 12.3	^2^ *J*	−14.2	−13.5	−13.4
**9**	^2^*J* = 12.3	^2^ *J*	−13.3	−11.8	−12.5
**10**	*J*_CH,Me_ = 7.9	*J*_CH,Me_	6.0	6.4	6.7
**11**	*J*_CH,Me_ = 7.2	*J*_CH,Me_	6.3	7.3	7.6
**12**	*J*_CH,Me_ = 7.2	*J*_CH,Me_	5.6	6.4	6.7

^1^ Only calculated *J* corresponding to experimental ones are indicated. ^2^ Experimental data are absolute values; n.d. = not detected.

## Data Availability

Not applicable.

## Supplementary Materials

The following supporting information can be downloaded at: https://www.mdpi.com/article/10.3390/md21100511/s1, Figure S1: Molecular structures of polyarsenicals **1**–**12**; Table S1: Calculated ^1^H-NMR chemical shifts of polyarsenicals **1**–**12** in chloroform by using combinations of methods, functionals and basis sets; Table S2: Calculated ^13^C-NMR chemical shifts of polyarsenicals **1**–**12** in chloroform by using combinations of methods, functionals and basis sets. Table S3: xyz Coordinates of geometry optimized structures of compounds **1**–**12**.
