# Supplementary material for: A Comprehensive Computational NMR Analysis of Organic Polyarsenicals including the Marine Sponge-Derived Arsenicins A–D and Their Synthetic Analogs
Source: Mar Drugs. 2023 Sep 27;21(10):511. doi: 10.3390/md21100511 (PMC10608599; doi:10.3390/md21100511)
Supplement: Supplementary file 1 [file marinedrugs-21-00511-s001.zip › marinedrugs-2603243-supplementary.pdf]

Supplementary Materials

A Comprehensive Computational NMR Analysis of Organic Polyarsenicals Including the Marine Sponge-Derived Arsenicins A-D and Their Synthetic Analogs

Andrea Defant * and Ines Mancini *

Laboratory of Bioorganic Chemistry, Department of Physics, University of Trento, Via Sommarive 14, I-38123 Trento, Italy

* Correspondence: andrea.defant@ex-staff.unitn.it (A.D.); ines.mancini@unitn.it (I.M.)

Contents

Table S1. Calculated ^{1}H-NMR chemical shifts of polyarsenicals **1-12** in chloroform by using combinations of methods (GIAO, CSGT), functionals (OLYP, M06-L, PBE1PBE, TPSSh**)** and the indicated basis sets; experimental values in $CDCl_3$ for comparison, in redline. For the numbering of H and C atoms, see structures in Figure S1.

Table S2. Calculated ^{13}C-NMR chemical shifts of polyarsenicals **1-12** in chloroform by using combinations of methods (GIAO, CSGT), functionals (OLYP, M06-L, PBE1PBE, TPSSh) and the indicated basis sets; experimental values in $CDCl_3$ for comparison, in redline. For the numbering of H and C atoms, see structures in Figure S1.

Figure S1. Molecular structures (energy minimized using C-PCM in chloroform, B1B95/6-311+G(3df,2pd) of polyarsenicals **1-12** evaluated in the NMR calculations.

Table S3. xyz Coordinates of geometry optimized structures of compounds **1-12.**

Table S1. Calculated ^{1}H-NMR chemical shifts of polyarsenicals **1-12** in chloroform by using combinations of methods (GIAO, CSGT), functionals (OLYP, M06-L, PBE1PBE, TPSSh**)** and the indicated basis sets; experimental values in $CDCl_3$ for comparison, in redline. For the numbering of H and C atoms, see structures in Figure S1.

			**OLYP**									
	**Atoms**	**Expt.**	**aug-cc-p-VDZ**		**aug-cc-p-VTZ**		**def2-TZVPP**		**pcSseg-2**		**x2c-TZVPPAll-s**	
			**GIAO**	**CSGT**	**GIAO**	**CSGT**	**GIAO**	**CSGT**	**GIAO**	**CSGT**	**GIAO**	**CSGT**
**1**	**H6 e H9**	2.42	2.36	2.16	2.27	2.30	2.13	2.03	2.23	1.91	2.19	2.04
	**H2 e H3**	2.23	2.17	1.97	2.09	2.10	1.93	1.84	2.02	1.74	1.99	1.86
	**H5 e H8**	1.37	1.28	1.09	1.18	1.20	1.00	0.91	1.10	0.80	1.04	0.91
**2**	**H2-H4-H13-H14**	1.85	1.88	1.66	1.75	1.78	1.60	1.48	1.69	1.35	1.64	1.46
**3**	**H12-H13**	2.71	2.78	2.55	2.64	2.66	2.52	2.38	2.60	2.26	2.57	2.38
	**H11-H14**	1.71	1.75	1.53	1.61	1.63	1.45	1.33	1.54	1.21	1.49	1.32
**4**	**H3-H6-H8**	3.11	3.11	2.89	3.03	3.04	2.92	2.81	3.01	2.69	2.99	2.83
	**H2-H5-H9**	1.53	1.50	1.32	1.41	1.44	1.25	1.16	1.34	1.05	1.27	1.15
**5**	**H2-H4**	1.40	1.12	0.86	0.98	1.02	0.79	0.83	0.933	0.67	0.93	0.83
	**CH_3**	1.59	1.62	1.50	1.56	1.58	1.43	1.46	1.56	1.37	1.52	1.45
**6**	**H14**	3.96	3.89	3.66	3.90	3.92	3.69	3.54	3.83	3.53	3.71	3.52
	**H12**	3.46	3.42	3.20	3.43	3.43	3.22	3.10	3.37	3.09	3.24	3.08
	**H10**	3.44	3.20	2.92	3.17	3.15	2.99	2.78	3.14	2.76	3.01	2.75
	**H15**	2.34	2.19	1.87	2.03	2.03	1.91	1.74	1.99	1.65	1.86	1.63
	**H13**	1.92	1.76	1.50	1.63	1.65	1.52	1.39	1.61	1.30	1.50	1.32
	**H11**	1.56	1.35	1.07	1.20	1.20	1.08	0.93	1.17	0.82	1.05	0.83
**7**	**H14**	3.50	3.43	3.19	3.41	3.43	3.24	3.43	3.38	3.08	3.27	3.10
	**H10**	3.27	3.07	2.81	3.04	3.02	2.88	3.01	3.02	2.66	2.91	2.68
	**H12**	2.99	2.95	2.74	2.95	2.94	2.77	2.97	2.91	2.62	2.79	2.64
	**H15**	2.02	1.79	1.52	1.64	1.66	1.55	1.70	1.63	1.30	1.48	1.28
	**H13**	1.44	1.21	0.98	1.09	1.11	0.98	1.18	1.07	0.78	0.95	0.81
	**H11**	1.29	1.01	0.76	0.87	0.89	0.76	0.93	0.85	0.53	0.71	0.54
**8**	**H11-H14**	3.53	3.28	3.01	3.25	3.26	3.08	2.91	3.20	2.88	3.09	2.87
	**H10-H15**	2.10	1.83	1.51	1.66	1.67	1.58	1.41	1.66	1.32	1.52	1.29
	**H12-H13**	1.49	1.22	0.91	1.06	1.06	0.98	0.86	1.02	0.77	0.97	0.80
**9**	**H3-H5**	2.10	1.67	1.61	1.82	1.82	1.76	1.67	1.86	1.56	1.73	1.59
	**H2-H6**	1.64	1.18	1.09	1.28	1.27	1.26	1.11	1.34	0.98	1.21	1.00
	**H8-H9**	1.29	0.88	0.82	1.02	1.00	0.94	0.88	1.05	0.78	0.91	0.80
**10**	**H13**	3.97	3.58	3.19	3.49	3.50	3.38	3.21	3.46	3.15	3.41	3.19
	**H12**	2.51	2.39	1.99	2.22	2.24	2.13	2.02	2.18	1.93	2.09	1.91
	**H_3C10**	2.12	2.35	2.19	2.31	2.34	2.25	2.08	2.32	2.00	2.28	2.10
	**H_3C11**	1.11	0.96	0.82	0.88	0.89	0.86	0.73	0.88	0.70	0.84	0.68
**11**	**H10, H19**	3.76	3.27	2.88	3.17	3.19	3.07	3.00	3.12	2.83	3.09	2.86
	**H_3C11, H_3C15**	1.07	0.90	0.77	0.82	0.84	0.80	0.66	0.81	0.63	0.77	0.63
**12**	**H12, H13**	2.61	2.53	2.12	2.37	2.38	2.28	2.17	2.32	2.11	2.25	2.08
	**H_3C10, H_3C11**	2.21	2.46	2.31	2.44	2.46	2.37	2.20	2.42	2.16	2.40	2.23

Table S1, contd.

	Atoms	Expt.	M06-L									
			aug-cc-p-VDZ		aug-cc-p-VTZ		def2-TZVPP		pcSseg-2		x2c-TZVPPAll-s	
			GIAO	CSGT	GIAO	CSGT	GIAO	CSGT	GIAO	CSGT	GIAO	CSGT
**1**	**H6 e H9**	2.42	2.18	2.51	2.09	2.63	2.02	2.47	2.02	2.25	2.02	1.92
	**H2 e H3**	2.23	1.95	2.28	1.86	2.40	1.78	2.24	1.79	2.02	1.77	1.69
	**H5 e H8**	1.37	1.18	1.53	1.07	1.62	0.98	1.46	1.02	1.23	0.96	0.90
**2**	**H2-H4-H13-H14**	1.85	1.79	2.10	1.67	2.21	1.59	2.02	1.59	1.82	1.56	1.45
**3**	**H12-H13**	2.71	2.46	2.85	2.38	2.96	2.35	2.78	2.40	2.60	2.37	2.21
	**H11-H14**	1.71	1.56	1.92	1.41	2.01	1.36	1.83	1.41	1.63	1.39	1.24
**4**	**H3-H6-H8**	3.11	2.74	3.15	2.71	3.30	2.71	3.16	2.75	2.97	2.75	2.61
	**H2-H5-H9**	1.53	1.31	1.70	1.16	1.79	1.11	1.63	1.19	1.42	1.13	1.04
**5**	**H2-H4**	1.40	1.17	1.47	1.04	1.62	1.05	1.52	1.06	1.30	1.02	0.97
	**C*H*$_3$**	1.59	1.57	1.99	1.58	2.07	1.49	1.97	1.48	1.85	1.50	1.44
**6**	**H14**	3.96	3.64	3.18	3.53	4.11	3.51	3.93	3.48	3.19	3.52	3.37
	**H12**	3.46	3.16	2.71	3.10	3.64	3.05	3.49	3.04	2.78	3.06	2.93
	**H10**	3.44	2.95	2.46	2.85	3.39	2.82	3.20	2.82	2.45	2.86	2.62
	**H15**	2.34	1.93	1.39	1.80	2.37	1.79	2.17	1.81	1.47	1.76	1.55
	**H13**	1.92	1.50	1.01	1.44	1.98	1.39	1.81	1.43	1.11	1.38	1.22
	**H11**	1.56	1.13	0.63	1.04	1.60	1.00	1.40	1.05	0.69	0.99	0.79
**7**	**H14**	3.50	3.16	3.53	3.07	3.66	3.07	3.52	3.06	3.31	3.08	2.94
	**H10**	3.27	2.82	3.13	2.72	3.27	2.71	3.10	2.73	2.93	2.73	2.51
	**H12**	2.99	2.69	3.06	2.63	3.19	2.60	3.06	2.63	2.87	2.61	2.50
	**H15**	2.02	1.51	1.84	1.39	1.99	1.40	1.82	1.43	1.64	1.35	1.19
	**H13**	1.44	0.96	1.32	0.89	1.46	0.86	1.32	0.92	1.14	0.85	0.72
	**H11**	1.29	0.79	1.14	0.70	1.28	0.66	1.10	0.74	0.93	0.65	0.49
**8**	**H11-H14**	3.53	3.06	2.56	2.95	3.51	2.94	3.33	2.92	3.15	2.95	2.74
	**H10-H15**	2.10	1.57	1.05	1.45	2.02	1.47	1.85	1.49	1.69	1.43	1.22
	**H12-H13**	1.49	0.99	0.49	0.91	1.47	0.88	1.32	0.92	1.19	0.86	0.72
**9**	**H3-H5**	2.10	1.61	1.95	1.60	2.15	1.59	2.05	1.68	1.84	1.59	1.46
	**H2-H6**	1.64	1.14	1.46	1.12	1.66	1.13	1.53	1.24	1.40	1.11	0.92
	**H8-H9**	1.29	0.86	1.19	0.85	1.39	0.83	1.31	0.95	1.10	0.83	0.73
**10**	**H13**	3.97	3.42	2.30	3.21	2.57	3.28	2.42	3.23	3.52	3.35	3.13
	**H12**	2.51	2.08	3.60	1.99	3.84	1.95	3.70	1.96	2.26	1.94	1.80
	**H_3C10**	2.12	2.30	2.71	2.22	2.80	2.24	2.61	2.23	2.45	2.30	2.09
	**H_3C11**	1.11	0.93	1.37	0.88	1.44	0.88	1.29	0.89	1.22	0.89	0.74
**11**	**H10, H19**	3.76	3.15	3.33	2.99	3.57	3.02	3.43	3.00	3.23	3.10	2.90
	**H_3C11, H_3C15**	1.07	0.87	1.32	0.84	1.39	0.83	1.22	0.83	1.14	0.88	0.75
**12**	**H12, H13**	2.61	2.15	2.41	2.13	2.69	2.10	2.56	2.12	2.41	2.15	2.03
	**H_3C10, H_3C11**	2.21	2.42	2.82	2.35	2.89	2.34	2.71	2.32	2.57	2.42	2.24

Table S1, contd.

	Atoms	Expt.	PBE1PBE									
			aug-cc-p-VDZ		aug-cc-p-VTZ		def2-TZVPP		pcSseg-2		x2c-TZVPPAll-s	
			GIAO	CSGT	GIAO	CSGT	GIAO	CSGT	GIAO	CSGT	GIAO	CSGT
1	H6 e H9	2.42	2.42	2.23	2.22	2.34	2.13	2.02	2.20	1.87	2.16	2.01
	H2 e H3	2.23	2.14	1.95	1.94	2.05	1.83	1.74	1.91	1.60	1.86	1.73
	H5 e H8	1.37	1.27	1.09	1.05	1.17	0.93	0.84	1.01	0.69	0.95	0.82
2	H2-H4-H13-H14	1.85	1.93	1.64	1.68	1.81	1.58	1.46	1.66	1.30	1.60	1.43
3	H12-H13	2.71	2.79	2.49	2.54	2.66	2.47	2.33	2.52	2.17	2.49	2.31
	H11-H14	1.71	1.69	1.40	1.43	1.56	1.33	1.21	1.40	1.05	1.34	1.18
4	H3-H6-H8	3.11	3.18	2.89	2.99	3.09	2.92	2.82	2.98	2.65	2.96	2.80
	H2-H5-H9	1.53	1.38	1.12	1.16	1.29	1.04	0.97	1.13	0.81	1.04	0.94
5	H2-H4	1.40	1.25	0.91	1.00	1.13	0.91	0.86	0.97	0.68	0.95	0.85
	C*H*$_3$	1.59	1.69	1.47	1.52	1.63	1.49	1.42	1.54	1.32	1.49	1.42
6	H14	3.96	3.76	3.44	3.61	3.72	3.47	3.31	3.55	3.23	3.47	3.27
	H12	3.46	3.33	3.03	3.20	3.29	3.06	2.93	3.15	2.85	3.06	2.89
	H10	3.44	3.17	2.81	2.99	3.07	2.88	2.67	2.96	2.58	2.88	2.62
	H15	2.34	2.17	1.78	1.91	2.01	1.83	1.65	1.89	1.52	1.78	1.54
	H13	1.92	1.74	1.41	1.52	1.63	1.44	1.30	1.52	1.17	1.41	1.22
	H11	1.56	1.42	1.05	1.17	1.27	1.08	0.91	1.16	0.77	1.04	0.82
7	H14	3.50	3.35	3.04	3.19	3.30	3.09	2.96	3.18	2.84	3.09	2.91
	H10	3.27	3.08	2.74	2.91	2.98	2.81	2.63	2.89	2.52	2.81	2.58
	H12	2.99	2.92	2.63	2.78	2.87	2.67	2.56	2.76	2.45	2.67	2.52
	H15	2.02	1.72	1.38	1.47	1.59	1.41	1.26	1.48	1.13	1.34	1.14
	H13	1.44	1.17	0.86	0.95	1.06	0.88	0.77	0.96	0.63	0.83	0.69
	H11	1.29	1.00	0.68	0.77	0.88	0.68	0.55	0.77	0.40	0.63	0.45
8	H11-H14	3.53	3.17	2.82	3.00	3.10	2.89	2.71	2.96	2.61	2.88	2.65
	H10-H15	2.10	1.84	1.44	1.57	1.67	1.52	1.34	1.58	1.21	1.46	1.22
	H12-H13	1.49	1.19	0.81	0.95	1.04	0.87	0.76	0.92	0.64	0.86	0.68
9	H3-H5	2.10	1.74	1.43	1.90	1.69	1.55	1.45	1.64	1.30	1.52	1.37
	H2-H6	1.64	1.34	0.99	1.46	1.23	1.15	0.98	1.21	0.83	1.09	0.87
	H8-H9	1.29	1.04	0.71	1.19	0.96	0.83	0.76	0.93	0.62	0.80	0.68
10	H13	3.97	3.57	3.18	3.34	3.46	3.26	3.09	3.29	2.98	3.28	3.04
	H12	2.51	2.42	2.03	2.16	2.27	2.08	1.97	2.11	1.84	2.04	1.86
	H_3C10	2.12	2.43	2.26	2.28	2.39	2.23	2.04	2.28	1.94	2.26	2.05
	H_3C11	1.11	1.04	0.90	0.87	0.97	0.85	0.70	0.87	0.67	0.83	0.68
11	H10, H19	3.76	3.27	2.80	3.35	3.15	2.96	2.78	2.98	2.68	2.96	2.72
	H_3C11, H_3C15	1.07	0.98	0.76	1.11	0.91	0.79	0.63	0.80	0.59	0.76	0.60
12	H12, H13	2.61	2.56	2.07	2.29	2.41	2.22	2.12	2.25	2.03	2.20	2.02
	H_3C10, H_3C11	2.21	2.54	2.25	2.39	2.51	2.34	2.16	2.38	2.09	2.37	2.18

Table S1, contd.

	Atoms	Expt.	TPSSh									
			aug-cc-p-VDZ		aug-cc-p-VTZ		def2-TZVPP		pcSseg-2		x2c-TZVPPAll-s	
			GIAO	CSGT	GIAO	CSGT	GIAO	CSGT	GIAO	CSGT	GIAO	CSGT
**1**	**H6 e H9**	2.42	2.38	2.18	2.28	2.31	2.18	2.08	2.26	1.94	2.20	2.06
	**H2 e H3**	2.23	2.14	1.95	2.05	2.07	1.93	1.85	2.01	1.72	1.96	1.84
	**H5 e H8**	1.37	1.28	1.08	1.16	1.19	1.03	0.95	1.12	0.81	1.04	0.93
**2**	**H2-H4-H13-H14**	1.85	1.90	1.69	1.76	1.80	1.65	1.54	1.73	1.39	1.67	1.50
**3**	**H12-H13**	2.71	2.77	2.55	2.63	2.65	2.53	2.41	2.60	2.27	2.56	2.38
	**H11-H14**	1.71	1.72	1.50	1.56	1.60	1.45	1.34	1.53	1.19	1.46	1.31
**4**	**H3-H6-H8**	3.11	3.12	2.90	3.02	3.04	2.94	2.85	3.02	2.70	2.98	2.83
	**H2-H5-H9**	1.53	1.44	1.25	1.33	1.36	1.19	1.13	1.29	0.98	1.19	1.09
**5**	**H2-H4**	1.40	1.26	0.99	1.12	1.17	1.03	0.98	1.08	0.82	1.06	0.96
	**C*H*$_3$**	1.59	1.64	1.52	1.57	1.59	1.53	1.47	1.58	1.37	1.31	1.46
**6**	**H14**	3.96	3.78	3.54	3.73	3.75	3.58	3.44	3.67	3.37	3.58	3.39
	**H12**	3.46	3.33	3.10	3.29	3.30	3.14	3.03	3.25	2.97	3.15	2.99
	**H10**	3.44	3.13	2.85	3.07	3.06	2.94	2.74	3.03	2.67	2.94	2.69
	**H15**	2.34	2.18	1.87	2.01	2.02	1.92	1.75	1.98	1.63	1.86	1.64
	**H13**	1.92	1.64	1.48	1.61	1.63	1.52	1.40	1.60	1.27	1.49	1.32
	**H11**	1.56	1.38	1.10	1.23	1.24	1.13	0.98	1.21	0.84	1.09	0.88
**7**	**H14**	3.50	3.34	3.11	3.28	3.30	3.16	3.05	3.27	2.95	3.17	3.00
	**H10**	3.27	3.02	2.76	2.95	2.95	2.84	2.68	2.94	2.58	2.85	2.62
	**H12**	2.99	2.88	2.67	2.84	2.84	2.72	2.63	2.83	2.52	2.72	2.58
	**H15**	2.02	1.75	1.49	1.60	1.62	1.53	1.39	1.59	1.26	1.45	1.26
	**H13**	1.44	1.19	0.96	1.07	1.09	0.98	0.89	1.07	0.76	0.94	0.80
	**H11**	1.29	1.00	0.76	0.87	0.89	0.77	0.65	0.86	0.52	0.72	0.55
**8**	**H11-H14**	3.53	3.18	2.91	3.11	3.13	2.99	2.82	3.08	2.74	2.98	2.77
	**H10-H15**	2.10	1.83	1.52	1.66	1.67	1.59	1.43	1.66	1.31	1.53	1.31
	**H12-H13**	1.49	1.23	0.93	1.08	1.09	1.00	0.89	1.05	0.78	0.98	0.82
**9**	**H3-H5**	2.10	1.81	1.57	1.75	1.76	1.69	1.61	1.79	1.46	1.67	1.52
	**H2-H6**	1.64	1.37	1.10	1.28	1.27	1.26	1.10	1.33	0.96	1.21	0.99
	**H8-H9**	1.29	1.06	0.81	0.99	0.99	0.93	0.87	1.04	0.75	0.90	0.80
**10**	**H13**	3.97	3.57	3.17	3.45	3.47	3.36	3.20	3.40	3.10	3.37	3.15
	**H12**	2.51	2.41	2.01	2.25	2.28	2.17	2.06	2.19	1.95	2.12	1.95
	**C10H$_3$**	2.12	2.38	2.22	2.33	2.35	2.27	2.09	2.32	2.00	2.30	2.11
	**C11H$_3$**	1.11	1.00	1.30	0.92	0.94	0.90	0.76	0.92	0.73	0.88	0.74
**11**	**H10, H19**	3.76	3.28	2.89	3.16	3.18	3.07	2.91	3.10	2.82	3.07	2.84
	**H$_3$C11, H$_3$C15**	1.07	0.95	0.82	0.87	0.88	0.85	0.70	0.86	0.66	0.82	0.67
**12**	**H12, H13**	2.61	2.54	2.13	2.38	2.40	2.30	2.20	2.34	2.13	2.26	2.10
	**H$_3$C10, H$_3$C11**	2.21	2.49	2.32	2.44	2.49	2.38	2.21	2.42	2.15	2.41	2.22

Table S2. Calculated ^{13}C-NMR chemical shifts of polyarsenicals **1-12** in chloroform by using combinations of methods (GIAO, CSGT), functionals (OLYP, M06-L, PBE1PBE, TPSSh) and the indicated basis sets; experimental values in $CDCl_3$ for comparison, in redline. For the numbering of H and C atoms, see structures in Figure S1.

			OLYP									
	**Atoms**	**Expt.**	**aug-cc-p-VDZ**		**aug-cc-p-VTZ**		**def2-TZVPP**		**pcSseg-2**		**x2c-TZVPPAll-s**	
			**GIAO**	**CSGT**	**GIAO**	**CSGT**	**GIAO**	**CSGT**	**GIAO**	**CSGT**	**GIAO**	**CSGT**
**1**	**C4 e C7**	23.05	28.32	25.98	27.26	26.74	25.50	22.65	27.16	26.46	25.55	24.88
	**C1**	17.03	26.44	23.79	24.82	24.24	23.15	20.74	24.51	24.02	23.00	22.54
**2**	**C1-C3**	30.60	33.62	31.73	33.34	32.74	31.72	28.40	33.50	32.57	31.79	30.87
**3**	**C1-C3**	23.71	30.36	28.51	29.61	29.21	28.10	25.26	29.80	29.01	28.16	27.33
**4**	**C1-C4-C7**	17.82	27.41	24.83	25.88	28.91	24.43	21.95	26.08	25.39	24.52	23.84
**5**	**C1-C3**	32.32	36.07	33.75	34.97	34.80	34.63	31.72	35.74	35.10	34,23	33.83
	**$\underline{C}H_3$**	10.29	11.46	13.39	12.35	13.04	11.71	11.47	12.61	12.38	12.83	12.49
**6**	**C9**	42.20	53.47	50.39	52.28	52.01	51.38	48.72	53.79	53.20	50.86	49.91
	**C7**	27.40	39.60	36.79	38.15	37.79	37.06	34.64	38.99	38.50	36.49	35.81
	**C2**	25.70	37.12	34.28	35.55	35.07	34.15	31.52	36.09	35.61	33.39	32.51
**7**	**C9**	45.14	53.10	50.26	52.25	52.00	51.34	48.61	53.86	53.27	50.99	50.18
	**C2**	33.58	42.46	39.89	41.60	41.21	40.30	37.62	42.60	42.05	39.86	39.08
	**C7**	31.40	40.61	37.87	39.48	39.14	38.49	36.00	40.48	39.93	38.06	37.46
**8**	**C2-C9**	38.90	49.26	46.35	48.03	47.75	46.89	47.96	49.34	48.70	46.35	45.39
	**C8**	8.10	19.76	16.83	16.71	16.43	15.91	17.71	16.75	16.53	15.11	14.44
**9**	**C1-C4**	45.3	53.60	53.90	52.69	52.48	52.46	49.93	54.85	54.30	52.05	51.21
	**C7**	32.5	42.57	42.95	41.43	41.17	41.14	38.82	43.06	42.66	40.61	40.06
**10**	**C7**	48.8	57.40	59.07	56.56	56.18	56.56	54.08	58.43	58.14	55.42	54.85
	**C1**	43.0	53.43	54.78	52.40	51.90	52.34	49.94	53.87	53.71	51.14	50.70
	**C11**	20.0	18.96	25.87	20.60	21.57	20.22	19.90	21.60	21.27	21.71	21.18
	**C10**	13.3	12.58	19.38	13.84	14.88	13.74	13.56	14.57	14.16	14.88	14.39
**11**	**C1, C7**	48.8	57.08	54.11	56.21	55.75	56.23	53.77	57.88	57.79	54.99	54.48
	**C11, C15**	19.8	18.68	21.08	20.35	21.37	20.00	19.69	21.48	21.18	21.47	20.96
**12**	**C1, C7**	42.5	53.17	49.88	52.12	51.61	51.97	49.58	53.40	53.44	50.86	50.39
	**C10, C11**	12.9	12.55	14.68	13.82	14.76	13.66	13.53	14.63	14.33	14.76	14.32

Table S2, contd.

			M06-L									
	**Atoms**	**Expt.**	**aug-cc-p-VDZ**		**aug-cc-p-VTZ**		**def2-TZVPP**		**pcSseg-2**		**x2c-TZVPPAll-s**	
			GIAO	CSGT	GIAO	CSGT	GIAO	CSGT	GIAO	CSGT	GIAO	CSGT
**1**	**C4 e C7**	23.05	23.50	22.58	19.93	21.81	20.57	19.59	20.75	21.26	19.04	18.91
	**C1**	17.03	21.58	20.60	17.62	19.37	18.11	17.57	18.12	18.81	16.40	16.41
**2**	**C1-C3**	30.60	28.24	27.68	25.51	27.23	26.30	24.79	24.50	31.07	24.60	24.35
**3**	**C1-C3**	23.71	25.46	24.71	19.11	23.83	22.53	20.71	23.15	23.83	21.43	20.84
**4**	**C1-C4-C7**	17.82	23.72	21.87	16.96	20.87	19.37	19.11	19.98	20.73	18.46	17.99
**5**	**C1-C3**	32.32	33.40	32.42	28.93	31.51	31.11	30.30	31.69	32.44	29.54	29.88
	**$\underline{C}H_3$**	10.29	14.81	17.54	16.26	16.71	14.32	16.23	14.66	16.76	14.41	14.93
**6**	**C9**	42.20	49.03	47.13	43.20	45.99	45.65	45.25	46.48	44.54	43.66	43.34
	**C7**	27.40	36.08	34.31	30.15	32.83	32.17	31.88	32.92	31.34	30.31	29.92
	**C2**	25.70	33.26	31.91	27.01	30.29	39.39	29.04	30.16	28.73	27.56	27.00
**7**	**C9**	45.14	47.69	46.18	42.37	45.34	44.97	44.41	45.99	47.03	43.13	42.83
	**C2**	33.58	37.58	36.36	31.69	35.27	34.44	34.00	35.46	36.40	32.90	32.37
	**C7**	31.40	35.81	34.49	30.30	33.48	32.75	32.37	33.75	34.64	31.12	30.68
**8**	**C2-C9**	38.90	45.01	43.13	38.86	41.89	41.51	41.00	42.26	43.20	39.62	39.09
	**C8**	8.10	16.69	14.66	10.24	12.53	11.43	11.37	11.51	12.46	9.62	9.27
**9**	**C1-C4**	45.3	49.59	47.7	44.70	46.9	46.96	46.6	48.17	49.2	45.07	44.61
	**C7**	32.5	39.04	37.3	34.11	36.3	36.18	36.0	37.32	38.1	34.34	33.96
**10**	**C7**	48.8	54.78	52.67	48.33	51.46	51.56	51.82	52.99	53.98	49.81	49.70
	**C1**	43.0	51.37	49.55	45.36	48.18	48.34	48.64	49.49	50.77	46.14	46.42
	**C11**	20.0	22.45	24.79	21.36	24.26	22.38	23.95	22.59	24.63	22.44	22.93
	**C10**	13.3	17.27	19.24	13.45	18.62	16.46	18.37	16.92	18.71	16.85	16.95
**11**	**C1, C7**	48.8	51.96	52.48	48.11	51.30	51.50	51.68	53.09	53.81	54.00	54.04
	**C11, C15**	19.8	20.28	24.64	22.36	24.08	22.08	23.76	22.93	24.41	25.14	25.76
**12**	**C1, C7**	42.5	51.77	49.13	45.91	47.71	48.32	48.12	49.24	50.29	49.89	50.27
	**C10, C11**	12.9	16.72	19.17	15.31	18.52	16.23	18.32	17.36	18.64	19.80	19.99

Table S2, contd.

	Atoms	Expt.	PBE1PBE									
			aug-cc-p-VDZ		aug-cc-p-VTZ		def2-TZVPP		pcSseg-2		x2c-TZVPPAll-s	
			GIAO	CSGT	GIAO	CSGT	GIAO	CSGT	GIAO	CSGT	GIAO	CSGT
**1**	**C4 e C7**	23.05	28.13	25.80	26.25	26.39	25.58	22.52	26.23	25.39	24.87	24.45
	**C1**	17.03	25.12	22.52	22.68	22.78	21.96	19.37	22.41	21.77	21.06	20.85
**2**	**C1-C3**	30.60	34.34	32.36	33.14	33.21	32.72	29.15	32.61	32.32	31.98	31.33
**3**	**C1-C3**	23.71	29.98	28.03	28.36	28.57	27.91	26.99	28.51	27.59	27.14	26.57
**4**	**C1-C4-C7**	17.82	26.46	23.73	24.01	24.29	23.43	20.81	24.07	23.29	22.74	22.35
**5**	**C1-C3**	32.32	36.84	34.38	34.97	35.34	35.05	32.21	35.58	34.78	34.18	34.15
	**$\underline{C}H_3$**	10.29	10.76	12.63	11.23	12.41	10.58	10.43	11.20	11.11	11.21	11.38
**6**	**C9**	42.20	52.19	49.10	50.17	50.51	50.01	47.23	51.31	50.51	48.78	48.15
	**C7**	27.40	38.27	35.49	36.06	36.27	35.69	33.13	36.71	36.02	34.41	33.97
	**C2**	25.70	36.55	33.72	34.28	34.36	33.64	30.84	34.69	33.95	32.21	31.50
**7**	**C9**	45.14	52.10	49.24	50.47	50.80	50.34	48.87	51.73	50.97	49.33	48.80
	**C2**	33.58	43.13	39.55	40.53	40.71	40.06	38.59	41.35	40.59	38.99	38.38
	**C7**	31.40	39.53	36.83	37.64	37.90	37.42	36.19	38.48	37.79	36.33	35.97
**8**	**C2-C9**	38.90	48.16	45.25	46.18	46.47	45.82	42.96	47.13	46.28	44.60	43.89
	**C8**	8.10	17.97	15.05	14.26	14.51	13.94	11.74	14.08	13.73	12.41	11.99
**9**	**C1-C4**	45.3	53.38	50.29	51.77	52.11	52.00	49.40	53.52	52.78	51.06	50.47
	**C7**	32.5	41.93	38.94	40.03	40.36	40.27	37.93	41.44	40.89	39.17	38.89
**10**	**C7**	48.8	57.01	53.94	55.44	55.62	55.59	53.26	56.75	56.21	54.10	53.80
	**C1**	43.0	52.30	48.94	50.42	50.49	50.56	48.44	51.40	51.29	48.91	48.91
	**C11**	20.0	19.28	21.41	20.45	21.87	20.14	19.90	21.00	20.92	21.10	21.20
	**C10**	13.3	12.41	14.43	13.17	14.61	12.93	12.80	13.44	13.19	13.56	13.59
**11**	**C1, C7**	48.8	56.71	53.63	55.18	55.28	55.31	53.03	56.31	56.10	53.73	53.51
	**C11, C15**	19.8	19.05	21.24	20.23	21.70	19.95	19.71	20.93	20.93	20.89	20.99
**12**	**C1, C7**	42.5	51.95	48.54	50.03	50.09	50.10	47.97	50.86	51.06	48.51	48.48
	**C10, C11**	12.9	12.37	14.29	13.07	14.46	12.81	12.73	13.48	13.41	13.41	13.48

Table S2, contd.

	Atoms	Expt.	TPSSh									
			aug-cc-p-VDZ		aug-cc-p-VTZ		def2-TZVPP		pcSseg-2		x2c-TZVPPAll-s	
			GIAO	CSGT	GIAO	CSGT	GIAO	CSGT	GIAO	CSGT	GIAO	CSGT
1	C4 e C7	23.05	26.72	24.46	25.42	24.93	24.72	21.74	25.42	24.61	23.97	23.53
	C1	17.03	24.26	21.72	22.44	21.88	21.74	19.21	22.21	21.60	20.79	20.53
2	C1-C3	30.60	32.50	30.59	31.85	31.30	31.36	27.87	32.11	31.07	30.57	29.91
3	C1-C3	23.71	28.63	26.80	27.60	27.22	27.14	24.17	27.82	26.95	26.33	25.76
4	C1-C4-C7	17.82	25.50	22.84	23.55	23.31	23.11	20.53	23.78	23.02	22.36	21.93
5	C1-C3	32.32	35.73	33.56	34.52	34.35	34.66	31.91	35.28	34.50	33.66	33.58
	$\underline{C}H_3$	10.29	11.68	13.60	12.89	13.45	12.28	12.14	12.90	12.81	12.95	13.06
6	C9	42.20	52.04	49.05	50.55	50.29	50.48	47.80	51.90	51.15	49.20	48.58
	C7	27.40	37.93	35.25	36.32	35.92	36.03	33.57	37.11	36.49	34.73	34.27
	C2	25.70	35.70	33.02	34.06	33.55	33.50	30.84	34.59	33.96	32.04	31.36
7	C9	45.14	51.63	48.89	50.53	50.26	50.46	47.68	51.97	51.25	49.39	48.85
	C2	33.58	41.01	38.58	40.01	39.59	39.58	36.84	40.95	40.27	38.46	37.86
	C7	31.40	38.85	36.27	37.57	37.31	37.40	34.85	38.53	37.88	36.27	35.86
8	C2-C9	38.90	47.84	45.04	46.37	46.08	46.10	43.36	47.53	46.75	44.85	44.15
	C8	8.10	17.66	14.83	14.59	14.29	14.42	12.33	14.58	14.26	12.92	12.46
9	C1-C4	45.3	53.05	50.16	52.01	51.79	52.32	49.81	53.96	53.27	51.31	50.70
	C7	32.5	41.52	38.73	40.27	40.03	40.61	38.30	41.86	41.33	39.44	39.10
10	C7	48.8	56.20	54.02	55.76	55.42	56.11	53.91	57.38	56.90	54.41	54.10
	C1	43.0	52.01	49.46	51.31	50.83	51.64	49.60	52.59	52.48	49.77	49.73
	C11	20.0	18.98	21.84	21.40	22.33	21.24	21.02	22.14	22.04	22.22	22.26
	C10	13.3	12.51	15.28	14.61	15.55	14.53	14.43	15.03	14.80	15.22	15.22
11	C1, C7	48.8	56.61	53.78	55.59	55.13	55.90	53.73	57.19	56.99	54.10	53.87
	C11, C15	19.8	19.39	21.67	21.24	22.16	21.06	20.84	22.25	22.20	22.02	22.06
12	C1, C7	42.5	52.23	48.99	50.92	50.38	51.12	49.08	52.18	52.33	49.33	49.25
	C10, C11	12.9	13.10	15.13	14.55	15.41	14.42	14.37	15.26	15.16	15.07	15.10

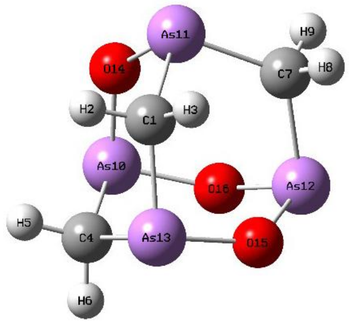

Arsenicin A (**1**)

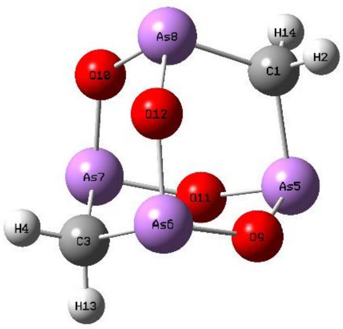

2

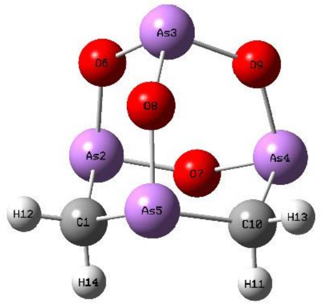

3

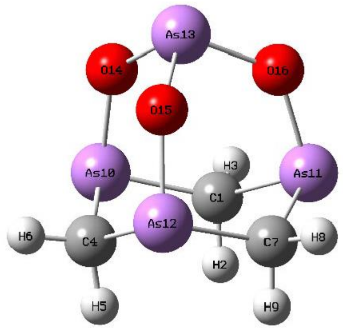

4

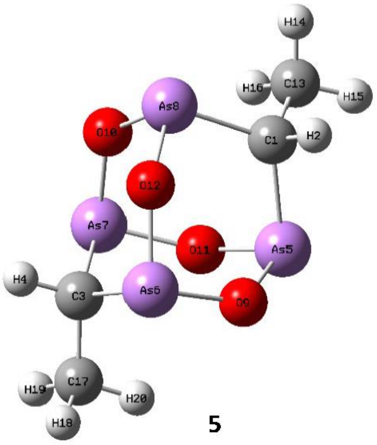

5

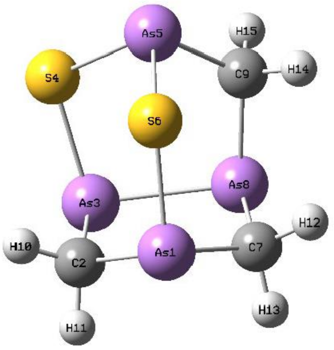

Arsenicin B (**6**)

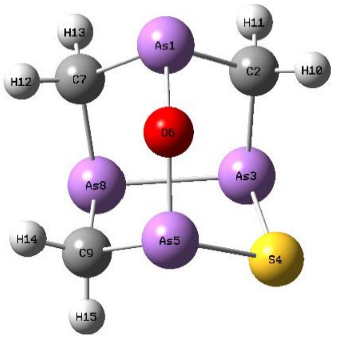

Arsenicin C (**7**)

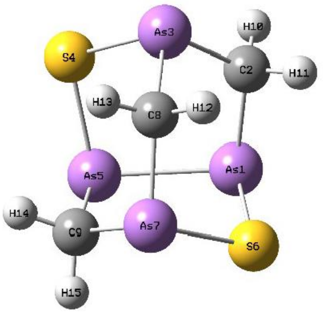

Arsenicin D (**8**)

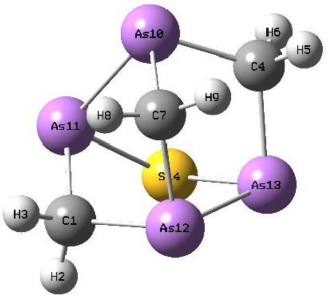

9

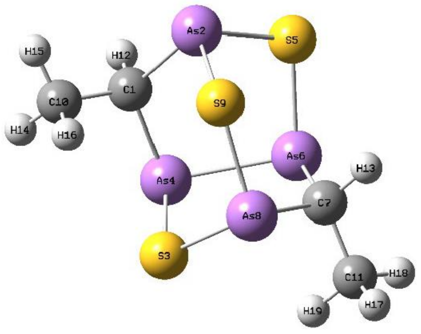

10

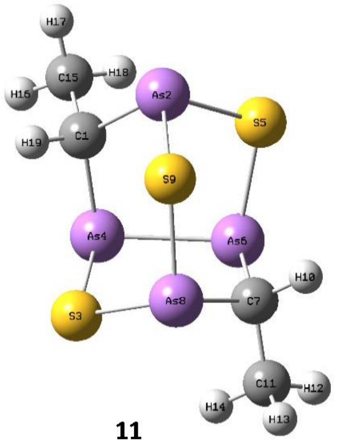

11

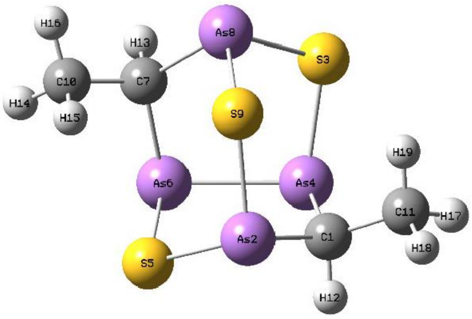

12

Figure S1. Molecular structures (energy minimized using C-PCM in chloroform, B1B95/6-311+G(3df,2pd) of polyarsenicals **1-12** evaluated in the NMR calculations.

Table S3. xyz Coordinates of geometry optimized structures of compounds **1-12**.

16

Arsenicin A (**1**)

C	0.00000	0.00000	-2.11395
H	-0.58715	0.65000	-2.76166
H	0.58715	-0.65000	-2.76166
C	0.00000	2.08822	0.00450
H	-0.63726	2.69567	-0.63857
H	0.57605	2.76996	0.62789
C	0.00000	-2.08822	0.00450
H	0.63726	-2.69567	-0.63857
H	-0.57605	-2.76996	0.62789
As	-1.19429	1.09604	1.19553
As	-1.26429	-1.14870	-1.15950
As	1.19429	-1.09604	1.19553
As	1.26429	1.14870	-1.15950
O	-1.97676	0.02490	0.00994
O	1.97676	-0.02490	0.00994
O	0.00000	0.00000	1.95466

……………………………………………………………

14

COMPOUND **2**

C	0.00000	-2.06517	0.00000
H	-0.62210	-2.70786	0.62210
C	0.00000	2.06517	0.00000
H	0.62210	2.70786	0.62210
As	-1.19861	-1.08662	-1.19862
As	-1.19861	1.08662	1.19862
As	1.19861	1.08662	-1.19862
As	1.19861	-1.08662	1.19862
O	-1.95987	0.00000	0.00000
O	1.95987	0.00000	0.00000
O	0.00000	0.00000	-1.95987
O	0.00000	0.00000	1.95987
H	-0.62210	2.70786	-0.62210
H	0.62210	-2.70786	-0.62210

……………………………………………………………

14

COMPOUND **3**

C	0.86176	-1.48452	-1.20073
As	-1.00799	-1.61647	-0.64833
As	-0.01971	-0.00113	1.96165
As	-1.00819	1.61710	-0.64648
As	1.97899	0.00049	-0.58318
O	-0.79925	-1.35789	1.11458
O	-1.57536	0.00057	-1.15826
O	1.56274	-0.00061	1.17867
O	-0.79937	1.35653	1.11612
C	0.86145	1.48588	-1.19930
H	0.86417	1.46062	-2.28912
H	1.34987	-2.40870	-0.89587
H	1.34952	2.40989	-0.89383
H	0.86475	-1.45840	-2.29052

……………………………………………………………

16

COMPOUND **4**

C	1.48723	0.85865	-1.23033
H	1.41373	0.81622	-2.31782
H	2.40600	1.38910	-0.99151
C	-1.48723	0.85865	-1.23033
H	-1.41373	0.81622	-2.31782
H	-2.40600	1.38910	-0.99151
C	0.00000	-1.71730	-1.23033
H	0.00000	-2.77820	-0.99151
H	0.00000	-1.63244	-2.31782
As	0.00000	1.97116	-0.61049
As	1.70708	-0.98558	-0.61049
As	-1.70708	-0.98558	-0.61049
As	0.00000	0.00000	1.96402
O	0.00000	1.57476	1.15415
O	-1.36378	-0.78738	1.15415
O	1.36378	-0.78738	1.15415

……………………………………………………………

20

COMPOUND **5**

C	0.00000	2.09141	0.05709
H	0.65385	2.67939	0.70344
C	0.00000	-2.09141	0.05709
H	-0.65385	-2.67939	0.70344
As	1.20931	1.07256	-1.11037
As	1.18022	-1.10359	1.27959
As	-1.20931	-1.07256	-1.11037
As	-1.18022	1.10359	1.27959
O	1.95917	-0.02025	0.08901
O	-1.95917	0.02025	0.08901
O	0.00000	0.00000	-1.87787
O	0.00000	0.00000	2.04135
C	-0.85959	3.00142	-0.81319
H	-1.48796	3.65496	-0.20867
H	-0.24727	3.63946	-1.44989
H	-1.51242	2.41616	-1.45848
C	0.85959	-3.00142	-0.81319
H	1.48796	-3.65496	-0.20867
H	0.24727	-3.63946	-1.44989
H	1.51242	-2.41616	-1.45848

……………………………………………………………

15

Arsenicin B (**6**)

As	-1.79614	1.22764	-0.30163
C	-1.41396	-0.21995	-1.55826
As	-0.16841	-1.64653	-1.00966
S	1.79646	-0.62379	-1.32885
As	1.89658	0.95103	0.24526
S	0.16746	2.30761	-0.17294
C	-1.74887	0.11742	1.30817
As	-0.38164	-1.28949	1.41496
C	1.19707	-0.15396	1.69780
H	-1.05271	0.19600	-2.49530
H	-2.37049	-0.70471	-1.75140
H	-1.63847	0.76617	2.17428
H	-2.72138	-0.36617	1.37623
H	0.97455	0.50540	2.53612
H	2.01711	-0.80649	1.98797

……………………………………………………………

15

Arsenicin C (**7**)

As	-1.48598	1.42222	-0.46585
C	-1.27010	-0.12908	-1.63152
As	-0.14501	-1.59893	-0.95513
S	1.85429	-0.61156	-1.17845
As	1.72350	1.07600	0.27878
O	0.22724	1.91609	-0.20599
C	-1.79387	0.35084	1.13681
As	-0.52922	-1.12412	1.43301
C	1.05411	-0.00909	1.75690
H	-0.86501	0.17910	-2.59231
H	-2.27034	-0.52873	-1.79936
H	-1.76658	0.99903	2.01065
H	-2.79838	-0.06137	1.06026
H	0.81026	0.66111	2.58160
H	1.87426	-0.64929	2.07216

……………………………………………………………

15

Arsenicin D (**8**)

As	-0.27434	-1.44051	1.20416
C	1.03296	-0.10218	1.81270
As	1.66984	1.26157	0.56924
S	2.09142	-0.14799	-1.10986
As	0.27434	-1.44052	-1.20416
S	-2.09142	-0.14799	1.10986
As	-1.66984	1.26157	-0.56924
C	0.00000	2.12292	0.00000
C	-1.03296	-0.10218	-1.81270
H	1.91593	-0.64240	2.14638
H	0.58315	0.38475	2.67704
H	-0.28542	2.77501	0.82494
H	0.28542	2.77502	-0.82494
H	-0.58315	0.38474	-2.67705
H	-1.91593	-0.64240	-2.14638

……………………………………………………………

14

COMPOUND **9**

C	0.00000	0.00000	-2.07563
H	-0.44596	0.76172	-2.71178
H	0.44596	-0.76172	-2.71178
C	0.00000	1.98162	-0.11090
H	-0.42938	2.61148	-0.89035
H	0.43086	2.61656	0.65814
C	0.00000	-1.98162	-0.11090
H	0.42938	-2.61148	-0.89035
H	-0.43086	-2.61656	0.65814
As	1.36699	0.87661	-0.97340
As	-1.42028	0.92219	0.74043
As	-1.36699	-0.87661	-0.97340
As	1.42028	-0.92219	0.74043
S	0.00000	0.00000	2.19055

……………………………………………………………

19

COMPOUND **10**

C	1.81299	0.02581	1.14406
As	2.10881	-0.17973	-0.79000
S	-1.18438	-1.19652	1.62314
As	0.04607	0.65717	1.79126
S	1.11312	1.70332	-1.42867
As	-0.77163	1.79438	-0.23184
C	-1.82961	0.32687	-1.03461
As	-1.39739	-1.53430	-0.57234
S	0.68868	-1.81934	-1.34080
C	2.25520	-1.22070	1.89644
C	-3.29128	0.55770	-0.66948
H	2.46471	0.86723	1.38550
H	-1.66748	0.41685	-2.10842
H	2.18376	-1.07387	2.97286
H	3.28969	-1.46834	1.66479
H	1.63172	-2.07114	1.63172
H	-3.93770	-0.18446	-1.13690
H	-3.62092	1.54203	-0.99797
H	-3.43973	0.49635	0.40751

……………………………………………………………

19

COMPOUND **11**

C	-1.77894	-0.12759	1.17095
As	-1.86316	1.21242	-0.26437
S	1.40609	-0.18535	1.94051
As	-0.28941	-1.43075	1.19859
S	-1.40610	-0.18535	-1.94051
As	0.28941	-1.43075	-1.19859
C	1.77894	-0.12759	-1.17095
As	1.86316	1.21242	0.26437
S	0.00000	2.42209	0.00000
H	1.68088	0.43276	-2.10039
C	3.08127	-0.91814	-1.13127
H	3.14050	-1.60832	-1.97131
H	3.94621	-0.25737	-1.17990
H	3.15852	-1.49899	-0.21342
C	-3.08127	-0.91814	1.13127
H	-3.14050	-1.60832	1.97131
H	-3.94621	-0.25736	1.17990
H	-3.15852	-1.49899	0.21342
H	-1.68088	0.43276	2.10039

………………………………………………………….

19

COMPOUND **12**

C	-2.05471	-0.54070	-0.21426
As	-1.66325	0.88409	1.08470
S	0.70169	-2.29496	-0.34354
As	-0.67719	-1.02394	-1.55911
S	-0.70169	2.29496	-0.34350
As	0.67721	1.02397	-1.55908
C	2.05471	0.54070	-0.21423
As	1.66324	-0.88411	1.08470
S	-0.00002	-0.00002	2.30083
C	2.59789	1.78011	0.48123
C	-2.59790	-1.78012	0.48116
H	-2.82969	-0.07962	-0.82887
H	2.82970	0.07963	-0.82883
H	2.90395	2.53321	-0.24313
H	1.84235	2.22245	1.12570
H	3.46668	1.53441	1.08950
H	-2.90395	-2.53321	-0.24322
H	-3.46670	-1.53443	1.08943
H	-1.84237	-2.22248	1.12563

………………………………………………………….
